# Effect of Mo and Ta on the Mechanical and Superelastic Properties of Ti-Nb Alloys Prepared by Mechanical Alloying and Spark Plasma Sintering

**DOI:** 10.3390/ma14102619

**Published:** 2021-05-17

**Authors:** Damian Kalita, Łukasz Rogal, Katarzyna Berent, Anna Góral, Jan Dutkiewicz

**Affiliations:** 1 Institute of Metallurgy and Materials Science, Polish Academy of Sciences, 25 Reymonta St., 30-059 Krakow, Poland; l.rogal@imim.pl (Ł.R.); a.goral@imim.pl (A.G.); j.dutkiewicz@imim.pl (J.D.); 2 Academic Centre for Materials and Nanotechnology, AGH University of Science and Technology, 30 Mickiewicza Av., 30-059 Krakow, Poland; kberent@agh.edu.pl

**Keywords:** titanium alloys, superelastic alloys, Ti-Nb alloys, twinning, deformation mechanisms, mechanical properties, powder metallurgy

## Abstract

The effect of ternary alloying elements (Mo and Ta) on the mechanical and superelastic properties of binary Ti-14Nb alloy fabricated by the mechanical alloying and spark plasma sintering was investigated. The materials were prepared in two ways: (i) by substituting Nb in base Ti-14Nb alloy by 2 at.% of the ternary addition, giving the following compositions: Ti-8Nb-2Mo and Ti-12Nb-2Ta and (ii) by adding 2 at.% of the ternary element to the base alloy. The microstructures of the materials consisted of the equiaxed β-grains and fine precipitations of TiC. The substitution of Nb by both Mo and Ta did not significantly affect the mechanical properties of the base Ti-14Nb alloy, however, their addition resulted in a decrease of yield strength and increase of plasticity. This was associated with the occurrence of the {332} <113> twinning that was found during the in-situ observations. The elevated concentration of interstitial elements (oxygen and carbon) lead to the occurrence of stress-induced martensitic transformation and twinning mechanisms at lower concentration of β-stabilizers in comparison to the conventionally fabricated materials. The substitution of Nb by Mo, and Ta caused the slight improvement of the superelastic properties of the base Ti-14Nb alloy, whereas their addition deteriorated the superelasticity.

## 1. Introduction

Metastable β-phase titanium alloys, containing only non-toxic alloying elements like Nb, Ta or Zr, are very promising candidates for Ni-free superelastic alloys for application in medicine. Particularly interesting are alloys from a binary Ti-Nb system due to their excellent biocompatibility, corrosion resistance and mechanical behavior similar to the human bone [1,2]. The superelasticity of those alloys arises from the reverse thermoelastic martensitic transformation that takes place between the body centered cubic (BCC) β parent phase and the orthorhombic α″-martensite [3]. The best superelastic properties in the Ti-Nb system were observed in the alloys containing about 26 at.% of Nb [4]. The calculations [5] show that the maximum transformation strain for the Ti-26Nb alloy reached about 3% and may be achieved in the [011] direction. This value for the randomly oriented polycrystalline material is even lower—about 2.3%. A small transformation strain at compositions showing superelasticity is one of the main drawbacks of the binary Ti-Nb alloys. Typical recoverable strains for the solution-treated (ST) alloys are in the range between 1 and 2.5% [5,6,7]. These values may be slightly improved by increasing the critical stress for slip deformation. Kim et al [5] shows that the formation of nanometric precipitations of ω-phase during the aging, in the temperature range 300–400 °C, may increase the recoverable strain up to about 4%.

The superelastic properties of the binary Ti-Nb alloys may be also enhanced by the use of ternary alloying elements [8,9,10]. These additions modify the crystal structure of the α″-martensite, which leads to the changes in both transformation strain and temperature [3]. The addition of all of the alloying elements, which have been reported up to today (e.g., Zr, Ta, Mo, Au, Pd, Pt, Sn, Al, Ga, Ge, O and N), decrease both the *M_s_* and the transformation strain in β-Ti alloys [11]. Among the β-stabilizers the most promising element is Mo due to it positive effect on the transformation strain. Mo reduces *M_s_* temperature three times more than Nb, however, the transformation strain decreases only 2.6 times. Therefore, the addition of Mo as a substitute of Nb, increase the transformation strain, while keeping the transformation temperature at the same level [8]. The favorable effect on the transformation strain was also reported for Pt, however, the extremely high cost of this element rather excludes it from the application in commercial alloys [9]. On the other hand, the β-eutectoid stabilizers, such as Cr, Mn, Fe or Cu do not possess the sufficient biocompatibility [12]. Ta shows a similar effect to Nb on the both transformation strain and temperature, however, it may enhance a critical stress for the plastic deformation as a result of the strong solid solution strengthening [13,14].

Although the superelasticity of Ti-Nb based alloys is well established for the materials obtained by casting, those fabricated using powder metallurgy (PM) did not receive significant attention so far. The attempts to fabricate the sintered β-Ti alloys showed that they do not possess superelastic properties, if they have the same compositions as the conventionally fabricated alloys for which the effect was previously reported [15,16]. Lai et al. [16] revealed that the elevated concentration of oxygen observed in the sintered materials resulted in the significant decrease of the martensitic transformation temperature in comparison to conventionally fabricated materials. Consequently, the superelastic properties were observed in sintered Ti-13Nb and Ti-11Nb alloys containing 2.5 and 4.0 at.% of oxygen, respectively [16,17]. Similarly, our previous studies on the spark-plasma sintered Ti-Nb alloys, showed that in order to obtain the superelastic properties, the concentration of Nb has to be reduced from the initial 26 to 14 at.% [18].

The further modification of the chemical composition of the developed binary Ti-14Nb alloy, by Mo or Ta, is likely to improve the functional properties of this alloy. The effect of those alloying elements was systematically studied for materials obtained by the casting, however in the case of materials obtained in the PM route, the data is limited. Taking this into account, the aim of this studies was to investigate the effect of Mo and Ta on the mechanical and superelastic properties of the binary Ti-14Nb alloy developed for PM technology. The materials were analyzed in two variants: when the 2 at.% of Mo and Ta was added to the Ti-14Nb alloy and when the Nb was substituted by 2 at.% of those elements, taking into consideration their effect on the transformation temperature. Nb decrease the Ms temperature by 40 °C/at.%, whereas Mo by 120 °C/at.% [3], therefore in order to maintain the transformation temperature at the same level Nb may be substituted by Mo in the 3:1 ratio. On the other hand, the effect of Ta on the Ms is similar as Nb therefore they may be substituted in 1:1 ratio. This resulting in the following compositions Ti-14Nb-2Mo, Ti-14Nb-2Ta, Ti-8Nb-2Mo and Ti-12Nb-2Ta.

## 2. Materials and Methods

### 2.1. Materials Fabrication

Elemental powders of Ti (<100 µm, 99.9%), Nb (<44 µm, 99.8%), Mo (2–5 µm, 99.95%) and Ta (<44 µm, 99.9%) were used as initial materials. The starting blends, with the total weight of 50 g, were prepared in such a way to obtain the following compositions: Ti-14Nb, Ti-14Nb-2Mo, Ti-14Nb-2Ta, Ti-8Nb-2Mo and Ti-12Nb-2Ta (in at.%). Mechanical alloying was carried out using Pulverisette 7 (Fritsch, Idar-Oberstein, Germany) planetary ball mill with a rotation speed of 150 rpm and cemented tungsten carbide container and 5 mm in diameter grinding balls containing 93.8 wt % of WC and 6.0 wt % of Co. The ball-to-power weight ratio was 10:1. Time of milling was 30 h. In order to avoid extensive oxidation of powders during synthesis, all the operations with powders were conducted in a glovebox under protective argon atmosphere (O_2_ and H_2_O  <  1 ppm). Powders were consolidated using the HP D5/2 spark-plasma sintering (SPS) system (FCT Systeme GmbH, Frankenblick, Germany) at 1300 °C for 30 min under 35 MPa pressure in an argon atmosphere. The compacts 20 mm in diameter and 8 mm high were obtained. The materials were next annealed at temperature 1250 °C for 24 h in a laboratory resistance furnace, in order to improve the chemical homogeneity, followed by the water quenching. To prevent oxidation during annealing, the samples were encapsulated in quartz tubes under vacuum. 

### 2.2. Materials Characterization

The phase composition of the prepared materials was examined by a PW1740 (Philips, Amsterdam, Netherlands) X-ray diffractometer (XRD) using Co-Kα radiation. The microstructural observations were carried out using scanning electron microscopes (SEM) Philips XL-40 (Amsterdam, Netherlands) and Versa 3D FEG (FEI, Hillsboro, OR, USA) equipped with an Apollo XP energy-dispersive X-ray spectrometer and Hikari CCD camera (EDAX, Berwyn, IL, USA) used for electron backscatter diffraction (EBSD) measurements. Backscattered electron (BSE) detector in Z-contrast was used with an acceleration voltage of 20 kV. More detailed analysis was performed using a Tecnai FEG G2 F20 Super Twin (FEI, Hillsboro, OR, USA) transmission electron microscope (TEM). Thin foils for TEM observations were prepared using a Tenupol-5 jet-polisher (Struers, Ballerup, Denmark) in an electrolyte consisting of 10 vol.% of H_2_SO_4_ in methanol at 10 °C. Mechanical properties of the investigated materials were analyzed by compressive tests using an Autograph AG–X plus (Shimadzu, Kyoto, Japan) testing machine at the strain rate of 10^−3^ s^−1^. In order to determine the superelastic properties, cyclic compressive tests were conducted. In the first cycle, compressive strain reached 1.5% and then the stress was removed. The tests were repeated for the same sample by increasing the strain by 0.5% for each cycle up to 5%. Three tests were carried out for each material. The value of the recoverable strain was calculated as a difference between the maximum strain and permanent strain occurred after the last cycle. Samples for the compression tests were prepared in the form of cylinders 4 mm in diameter and 6 mm in height using electrical discharge machining. The in-situ deformation experiments were performed using home-made tensile stage for a sample with the gauge length of 8 mm, width of 2 mm and 300 µm thick. The microstructure was observed using DMIRM (Leica, Wetzlar, Germany) optical microscope (OM). The oxygen content in prepared materials was determined by an inert gas fusion technique using an ON836 analyzer (Leco, St. Joseph, MI, USA).

## 3. Results and Discussion

### 3.1. Powders Characterization 

**Figure 1:**
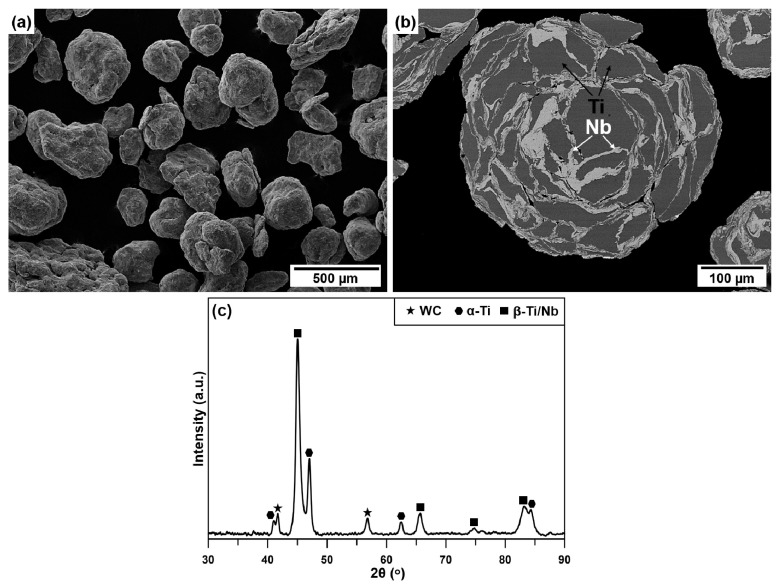
Typical SEM/SE (**a**) and SEM/BSE (**b**) micrographs of the powders after the MA on the example of the Ti-14Nb alloy and XRD pattern of this material (**c**).

Figure 1a shows a typical secondary electron (SE) SEM image of the mechanically alloyed powders prepared for the sintering on the example of binary Ti-14Nb alloy. The particles possessed the near spherical morphology with the grain size in the range of 100–400 µm. As resulted from the BSE image (Figure 1b), they exhibited lamellar internal microstructure in which the brighter layers enriched in Nb may be observed between the darker areas enriched in Ti. This observation is in agreement with the phase composition analysis by XRD (Figure 1c). The powder contains the mixture of α-Ti and β-Ti/Nb phases. β-Ti and Nb crystalize in the BCC structure in the space group Im-3m, with a slight difference in lattice parameters (*a_β-T_* = 3.327 Å and *a_Nb_* = 3.305 Å), so it is difficult to distinguish them using the XRD technique. One may notice, that the XRD pattern of the material contain also the weak peaks, which correspond to the tungsten carbide (WC). The occurrence of this phase in the powders after the MA is associated with the use of the milling balls made of tungsten cemented carbide. Due to a wear process that take place during the milling, the WC phase was introduced to the particles. As described in our previous work [18] the amount of the carbides increases with the increasing milling time, therefore the intermediate synthesis time of 30 h was selected in order to avoid the extensive contamination and to obtain a satisfactory level of the mixing of the elemental powders. 

### 3.2. Phase Composition and Microstructure

**Figure 2:**
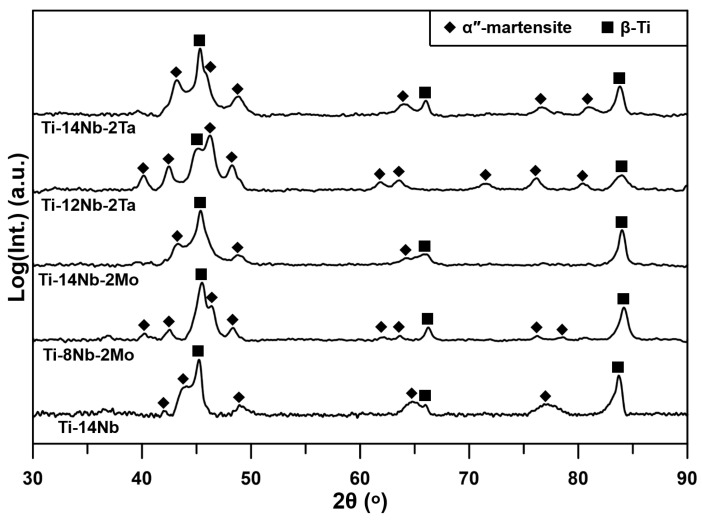
XRD patterns of the investigated materials.

**Figure 3:**
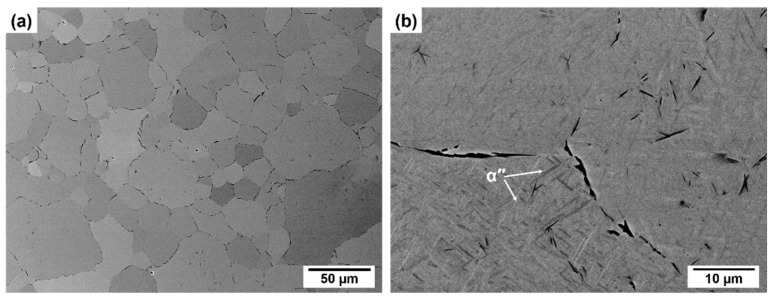
Typical SEM/BSE microstructure of the investigated materials on example of Ti-8Nb-2Mo alloy at lower (**a**) and higher (**b**) magnification.

Figure 2 shows XRD patterns of the investigated materials. All of them contain a mixture of BBC β-phase and α″-martensite. In the case of ternary Ti-Nb-Mo/Ta alloys, the intensities of α″-peaks decreased with the increasing Nb concentration, which is associated with the increasing stability of parent phase with the β-stabilizers content. The typical microstructure of the fabricated materials is shown in Figure 3. The microstructure of the materials consists of the equiaxed β-phase grains and precipitations depleted in alloying elements occurring at the grain boundaries and inside them. At higher magnifications, the extremely fine martensite laths with the width below 1 µm may be observed for all alloys. Their fraction was the lowest in the case of Ti-14Nb-2Mo alloy, which is in agreement with the XRD results. All alloys exhibited high relative density of 99.5% and relatively small grains with the average size of about 30 µm. This indicates the positive effect of application of mechanical alloying in the preparation of materials, since typically much larger grains were observed in the Ti-Nb-based alloys sintered from the elemental powders blends [15,19,20], e.g., Terayama et al. [15] reported that the relatively large grains, with an average size above 200 μm, resulted in a brittleness of the sintered Ti-22Nb alloy. The oxygen concentration measured in the materials using the IGF technique was independent of their chemical composition and reached 0.7 ± 0.1 at.%. This value is one of the lowest observed for the sintered β-Ti alloys, since typically the concentrations in the range of 1–4 at.% were reported in the literature [16,17,21].

**Figure 4:**
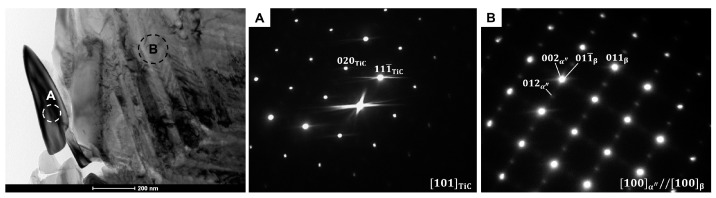
BF/TEM micrograph of Ti-8Nb-2Mo alloy presenting the elongated precipitation occurred inside the β-phase grain with the corresponding SADPs (A and B correspond to the areas marked by the circles).

**Figure 5:**
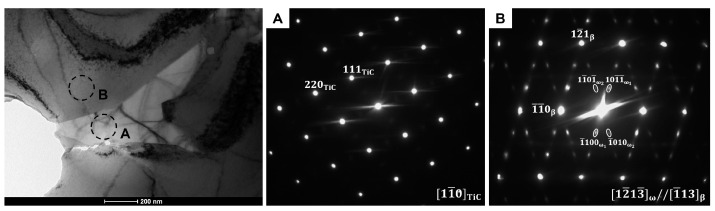
BF/TEM micrograph of Ti-14Nb-2Mo alloy presenting the precipitation occurred at the grain boundary with the corresponding SADPs (A and B correspond to the areas marked by the circles).

In order to identify the type of the precipitations observed in BSE/SEM images the TEM technique was applied. Figure 4 shows bright-field (BF) micrograph of one of the elongated precipitations occurring inside the β-phase grains in the Ti-8Nb-2Mo alloy. The selected area diffraction pattern (SEDP) registered from the marked area of the precipitation may be well indexed as TiC carbide with a ${\left[101\right]}_{\mathrm{TiC}}$ one axis. On the other hand, the SEDP taken from the region of the matrix indicates the coexistence of the parent β-phase and the α″-martensite, with the following orientation relationship: $[100{]}_{\;\alpha \;\prime \prime }//[100{]}_{\beta }$. Figure 5 presents BF image of one of the precipitations that may be observed at the grain boundaries in the Ti-14Nb-2Mo alloy. As in the previous case, the SEDP from the area of precipitation may be indexed as the TiC-phase with the ${\left[1\bar{1}0\right]}_{\mathrm{TiC}}$ zone axis. In the case of SEDP registered from the area of the matrix, in addition to the primary refractions from the β-phase with the ${\left[\bar{1}13\right]}_{\beta }$ zone axis, diffused scattering at 1/3 and 2/3 ${\left\{1\bar{2}1\right\}}_{\beta }$ may be observed. Those reflections are associated with the hexagonal ω-phase. Although the ω-phase non-equilibrium at an ambient condition (occurs at high pressures) it typically appears in the form of very fine precipitations in various metastable β-Ti alloys [22,23]. Its formation involves the collapse of a pair of ${\left(222\right)}_{\beta }$ planes to an intermediate position, which results in four crystallographic variants of ω-phase [3]. In this case, the presented SADP shows only two variants (ω_1_ and ω_2_), while the other two contribute to the β-refractions.

The occurrence of the precipitations of TiC carbides resulted from the presence of the WC phase in the powders after the mechanical alloying, as described in Section 3.1. During the sintering and annealing the WC particles reacted with the matrix forming TiC carbides. A similar effect has been utilized in the fabrication of in-situ reinforced metal matrix composites (MMC); e.g., Liu et al. [24] have demonstrated the ability to fabricate TiC strengthened titanium alloys by the reaction of Ti with Cr_3_C_2_ during the sintering. Li et al. [25] obtained Ti/TiC-TiB composite featuring superior strength by the sintering of a mixture of B_4_C and Ti powders. Although carbon exhibits strong tendency to formation of carbides it is important to note that it also possesses a slight solubility in β-Ti of about 1 at.% in pure Ti and of about 0.6 at.% in Ti-22Nb at the annealing temperature (1250 °C) [26].

### 3.3. Mechanical Properties

**Figure 6:**
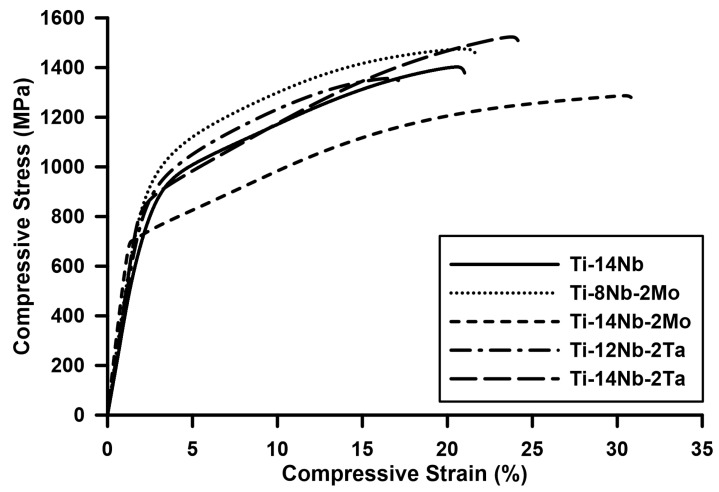
Compression stress–strain curves of the investigated materials.

In order to determine the mechanical properties of the investigated materials the compression tests were carried out. Figure 6 shows the representative compression stress-strain curves and Table 1 summarizes the obtained results. The substitution of Nb by Mo or Ta did not have a pronounced effect on the mechanical properties of the materials. The yield strength (YS) of the binary Ti-14Nb and ternary Ti-8Nb-2Mo and Ti-12Nb-2Ta alloys were close to 800 MPa. Similarly, the values of compressive strength and maximum compressive strain were comparable. On the other hand, the addition of ternary alloying elements to the base Ti-14Nb alloy resulted in the decrease of the YS and increase of the plasticity of the materials. However, the addition of Mo had a more pronounced effect on the YS in comparison to Ta, since resulted in drop of YS to 685 ± 15 MPa in Ti-14Nb-2Mo. The value for Ti-14Nb-2Ta was 766 ± 32 MPa.

The obtained values of YS are higher than those reported in the literature for the cast, binary Ti-Nb alloys, e.g., YS of the superelastic Ti-26Nb (most often studied) reach 200–400 MPa in solution-treated state [5,27] and 400–500 MPa after the aging [7,28]. The lower YS was also reported for ternary Ti-Nb-Mo/Ta alloys, e.g., 450 MPa for Ti-18Nb-3Mo [8] or 360 MPa for Ti-22Nb-5Ta [14]. In this case the higher YS in comparison to the conventionally fabricated Ti-Nb-based alloys resulted from the combination of the solid-solution strengthening effect caused by the interstitial elements (oxygen and carbon) and the occurrence of the TiC precipitations. As was reported by Kim et al. [29] oxygen highly affected the mechanical properties of Ti-Nb alloys—the YS of Ti-22Nb alloy increased from 350 MPa for the oxygen-free alloy to 1000 MPa for alloy containing 2 at.% of oxygen. Similar strengthening effect was also reported for carbon [30]. Additionally, the occurrence of the TiC precipitations in Ti/TiC composites typically resulted in very high YS, e.g., Zhang et al. [31] showed the increase of YS from an initial 1100–1700 MPa in the case of sintered Ti containing 3 vol.% of TiC (formed during the sintering of Ti powder with graphene). On the other hand, YS higher than 1000 MPa were typically registered for the Ti-Nb-based alloys obtained by the powder metallurgy [16,17,32]. This was mainly attributed to the very high oxygen contents observed in those materials, e.g., Yuan et al. [17] reported an extremely high YS of 1480 MPa in the sintered Ti-11Nb containing 4 at.% of O. However, the high strength was typically associated with the brittle behavior [17,33]. In this case, the developed fabrication method allowing for the reduction of oxygen content to 0.7 ± 0.1 at.%, resulting in their relatively high plasticity when compared with other materials obtained by the PM method, despite the occurrence of the strengthening TiC precipitations.

The positive effect of the ternary alloying elements on the YS of Ti-Nb alloys was previously reported in the literature, e.g., Al-Zain et al. [8] show that the substitution of Nb by Mo resulted in the increase of YS from 380 (Ti-27Nb) to 450 MPa (Ti-18Nb-3Mo). This was associated with the strong solid-solution strengthening effect caused by this element [13]. In this case, the effect of the substitution of Nb by Mo and Ta was unnoticeable. However, as described earlier the investigated materials exhibited significantly higher YS in comparison to the conventionally fabricated materials as a result of the elevated concentration of interstitial elements. The high lattice distortions introduced by the occurrence of those atoms may diminish the effect of the substitutional atoms. This allows one to state that the mechanical properties of the Ti-Nb-based alloys prepared by the PM route are mainly controlled by the concentration of the interstitial elements. On the other hand, even the drop in values of YS was observed, when Mo and Ta (2 at.% of each) were added to the base Ti-14Nb. Zhang et al. [13] show that also the slight addition of ternary elements may enhance the mechanical properties of the binary Ti-Nb alloys, e.g., the addition of 1 at.% of Mo to the binary Ti-22Nb alloy resulted in the increase of YS from 400 to 450 MPa. Another behavior was observed in other works in which the drop of the YS associated with the appearance of the twinning deformation mechanism with the increasing concentration of β-stabilizers was occurred [34,35]. However, the twinning was previously registered for the more heavily stabilized β-Ti alloys, e.g., Ti-27Nb [36] or Ti-23Nb-2Zr-1Ta [37]. Therefore, in order to explain the changes of the mechanical properties of the investigated materials the evolution of their microstructures at an early stage of the deformation was investigated in the more detail.

### 3.4. Microstructure Changes during the Deformation

**Figure 7:**
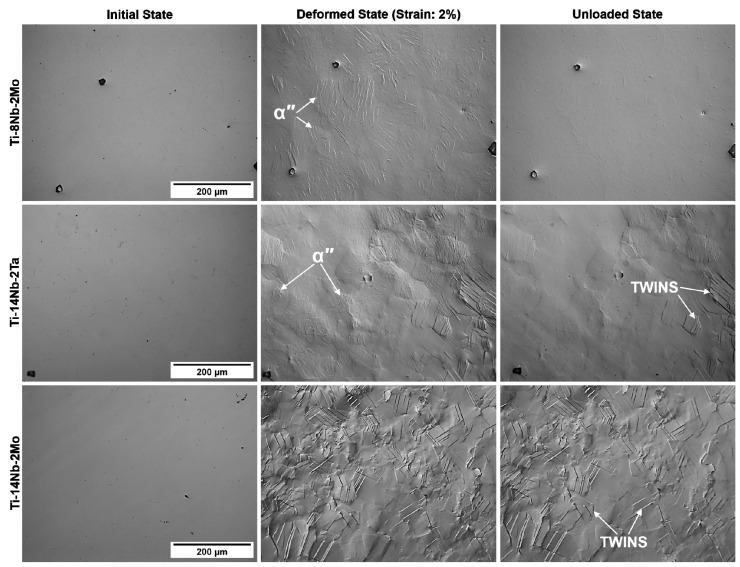
The OM microstructures of the investigated materials in the initial state, deformed to ε = 2% and when the stress was removed (unloaded state).

The deformation mechanism of the metastable β-Ti alloys is complex and highly dependent on the β-phase stability that is a function of the composition of the alloy. In general, with the increasing content of β-stabilizers, the deformation mechanisms follow the sequence: α″-martensite deformation → SIMT (β→α″) → twinning → slip [36,38]. Due to that the determination of the mechanisms that take place during the deformation of the analyzed materials may be crucial in order to find the origin of the changes in the values of YS after the addition of the ternary alloying elements. Figure 7 presents the optical microscope (OM) microstructures registered during the in-situ deformation experiments. The materials were investigated in the initial state, deformed to ε = 2% (in tensile mode) and after the stress was removed (unloaded state). In the case of Ti-8Nb-2Mo, the loading resulted in the formation of fine laths inside the β-phase grains, which totally disappeared when the stress was removed. This allows one to conclude that the first stage of the deformation of this material was controlled by the stress-induced martensitic transformation (SIMT) mechanism. Due to the fact that the mechanism is reversible, the favorable martensite variants formed during the loading are subjected to the reverse transformation during unloading. Although in Figure 7 the results for Ti-8Nb-2Mo are presented, the similar behavior was observed also for binary Ti-14Nb and ternary Ti-12Nb-2Ta alloys. Slightly different mechanism was registered for the Ti-14Nb-2Ta alloy. In this case, two different deformation mechanism coexist—the aforementioned SIMT and twinning. It is clearly visible that in the microstructure of the deformed sample fine martensite laths and larger twin bands are present. Next, during the unloading the majority of the laths disappear, but the twins remain unchanged. On the other hand, no signs of the reversible SIMT mechanism may be observed for Ti-14Nb-2Mo, which allows one to state that the contribution of this mechanism during the deformation was slight.

Although the occurrence of both SIMT and twinning mechanisms were previously reported in the literature for Ti-Nb-based alloys, they were typically observed in materials with higher concentrations of β-stabilizers. In the case of binary Ti-Nb alloys the SIMT was revealed in alloys with the Nb content higher than 26 at.%, whereas twinning in alloys containing of about 27–32 at.% of Nb [36,39,40]. In this case the SIMT was observed in binary Ti-14Nb alloy, and in ternary alloys in which the Nb was substituted by Mo or Ta according the effect of those elements on the transformation temperatures. Those alloys possessed similar transformation temperatures and similar mechanical properties. On the other hand, the drop of the YS was observed for Ti-14Nb-2Ta and Ti-14Nb-2Mo, which was attributed to the to the appearance of the twinning mechanism. This is in agreement with the literature, since the alloys in which twinning was observed typically display the low YS in the range of 400–500 MPa, e.g., 480 MPa for Ti-6.5Mo [35], high work-hardening rate and large uniform deformation [41,42]. Although both the Ti-14Nb-2Mo and Ti-14Nb-2Ta alloy contained the 2 at.% of ternary addition the coexistence of SIMT and twinning mechanisms were observed for the latter. This indicates that the effect of Mo addition of the β-phase stability is greater in comparison to this caused by Ta. Nevertheless, the presence of the aforementioned mechanisms at a lower concentration of β-stabilizers in the investigated materials may result from the positive impact of the interstitial elements at the β-phase stability. Both oxygen and carbon are recognized as the α-stabilizers, however the recent studies shows that those elements dissolute in the β-phase may also increase the stability of this phase similarly to the β-stabilizers [43]. 

**Figure 8:**
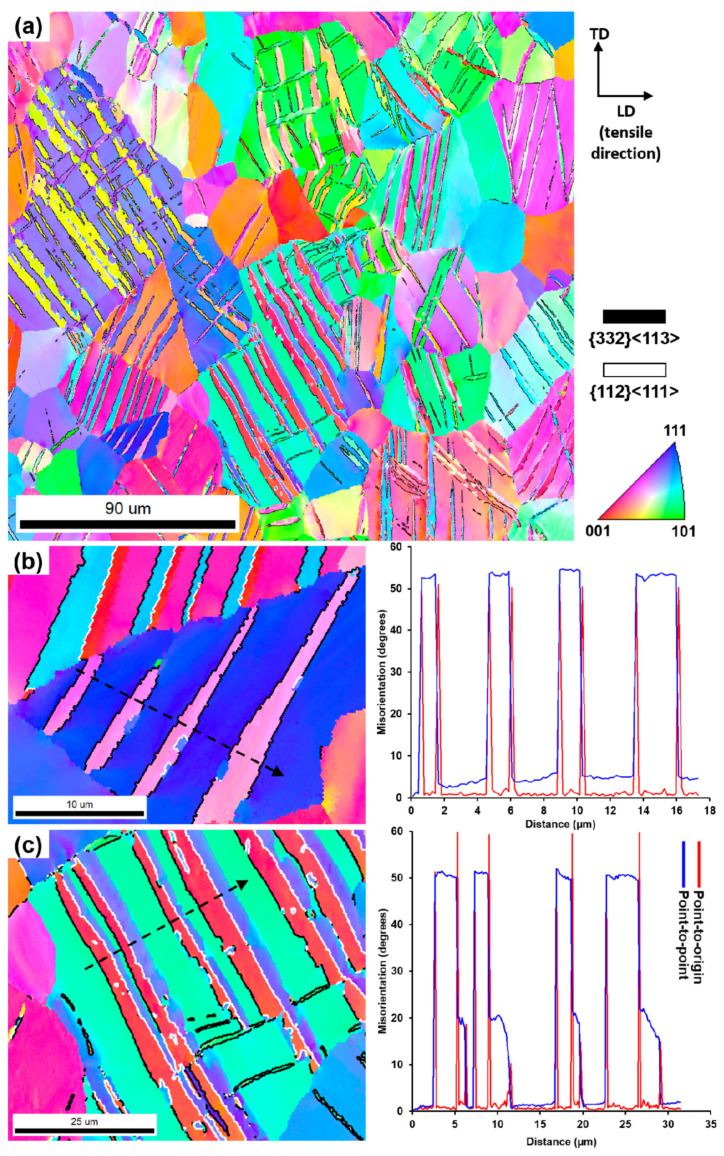
IPF map of the deformed Ti-14Nb-2Mo alloy (**a**) and selected grains at higher magnification (**b**,**c**) with the corresponding misorientation profile. The {332} <113> and {112} <111> twins boundaries are indicated by the black and white lines, respectively.

Figure 8 shows an inverse pole figure (IPF) map of the Ti-14Nb-2Mo alloy deformed up to ε = 2%. In its microstructure the twinning bands, several micrometers in thickness may be observed within the β-phase grains. Some of them have been identified as {332} <113>, with the characteristic 50.5° misorientation angle as shown in Figure 8b. However, the majority of bands were composed of two different parts, which varied in crystallographic orientations, as shown in Figure 8c. The point-to-point misorientation analyzed across the arrow in Figure 8c showed that the misorientation between the matrix and ‘red’ band varied from 49 to 51°, which corresponded to the {332} <113> twinning system. On the other hand, the misorientation angle between the ‘red’ band and adjacent ‘blue’ band reached about 60°, corresponding to the {112} <111> twinning system [44,45]. Next, the misorientation angle between the ‘blue’ band and the matrix was of about 15–20°, which did not correspond to any twinning system previously observed in β-Ti alloys. Similar phenomenon was also observed by Lai et al. [45] in the Ti-23Nb-2Zr-1Ta alloy. They found that the formation of the {332} <113> twins were accompanied with the formation of α”-martensite bands present only near the surface of the sample adjacent to the twin. Those bands may be next indexed as BCC β-phase during the measurement, since the α″ crystal structure may be derived from the β lattice by $\left\{0\bar{1}1\right\}\langle 011\rangle$ shuffle accompanied with a small shape change [46]. As a result, the EBSD Kikuchi patterns taken from the area of band may be indexed as both α″ or β phases with the same number of votes [45]. The {112} <111> twinning mode is most widely observed in the BCC metals, e.g., α-Fe, Nb and Mo [47]. However, in the case of conventionally fabricated β-Ti alloys, the less common {332} <113> system predominates [37,39,48]. Such alloys are recently extensively studied as a new group of β-Ti alloys with excellent deformability as a result of the twinning induced plasticity (TWIP) effect [42]. On the other hand, despite the promising capabilities of the PM methods in the fabrication of elements from the β-Ti alloys, the deformation behavior of those materials did not receive significant attention in the literature. The results obtained within the presented work show that the TWIP effect may be also obtained for the sintered Ti-Nb-based alloys.

### 3.5. Superelastic Properties

**Figure 9:**
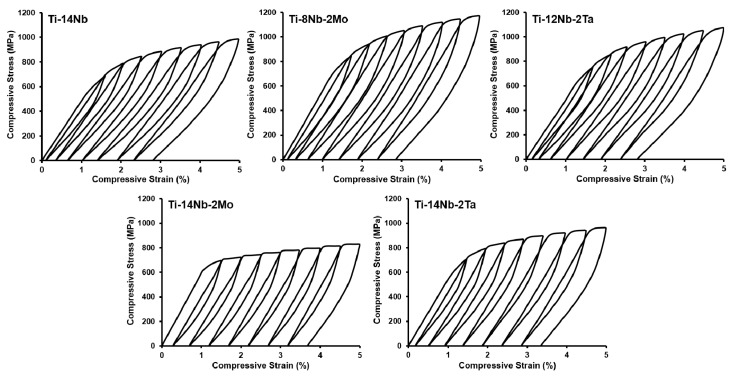
Cyclic compression stress–strain curves of the investigated materials.

The cyclic compressive tests were applied to study the superelastic properties of the fabricated materials. The measurements were carried out with increasing strain by 0.5%, starting with 1.5–5%. The tests were performed at room temperature. Figure 9 shows the representative cyclic compression stress–strain curves whereas the calculated recoverable strains were summarized in Table 2. 

The measured recoverable strain of the base Ti-14Nb alloy reached 2.1% ± 0.1%. This value is similar to those reported for the cast Ti-Nb alloys in the solution-treated state, e.g., Kim et al. [5] reported 2.5% of recoverable strain in Ti–26Nb. Lower values of 2.0 and 1.6% were showed for the same alloy by Kim et al. [7] and Zhang et al. [6], respectively. The higher recoverable strains were registered for the sintered Ti-Nb alloys, however enhanced superelastic properties were typically associated with the combination of high elasticity and high strength, e.g., 5.4% of recoverable strain was reported for Ti-11Nb alloy exhibited extremely high strength of 1480 MPa and low elastic modulus of 24.5 GPa [17]. The substitution of Nb by Mo or Ta did not have a pronounced effect of the superelasticity of the material, since the recoverable strain for those alloys was 2.2 ± 0.1%. On the other hand, the positive effect of those elements on the superelastic properties of binary Ti-Nb alloys was previously reported in the literature, e.g., Al-Zain [8] showed that the maximum recoverable strain of Ti-27Nb increased from 2.3 to 3.4% when the Nb was substituted by 3 at.% of Mo (Ti-18Nb-3Mo). Similarly, the increase of the recoverable strain from 2.0% for Ti-27Nb to 3.2% for Ti-21Nb-2Mo was reported by Kim et al. [3]. This result from the favorable effect of this element on the martensitic transformation strain and the critical stress for plastic deformation [3]. In the case of Ta, its effect of the transformation strain is similar as for Nb, but the positive impact on the critical stress for plastic deformation also resulted in the improvement of the superelasticity of the binary Ti-Nb alloys, e.g., recoverable strain of 3.2% was reported for Ti-22Nb-4Ta [14]. In this case, the slight effect of those elements may result from the non-conventional mechanism of superelasticity that is observed for these materials.

Typically, during the deformation of shape memory alloy in the austenitic state, a two-stage yielding is observed, the first yielding point is associated with the critical stress for the martensitic transformation and the second one with the critical stress for the plastic deformation [11]. Between these two points the characteristic stress-plateau occurs, where the superelastic deformation takes place [49]. In the case of the investigated materials only one-stage yielding was registered, despite it showing a superelastic response. Similar behavior was also observed in other works concerning the Ti-Nb based alloys containing raised concentrations of the interstitial elements [29,50]. This non-conventional mechanism is associated with the suppressing effect of the interstitial atoms on the SIMT. Salloom et al. [43] demonstrated that the oxygen atoms located in the octahedral sites form the stress fields that increase the energy required for the transformation. As a result, the values of the critical stress for the martensitic transformation and the critical stress for the plastic deformation (YS) are similar to those materials as reported by Castany et al. [51]. Taking into consideration the strong solid-solution strengthening effect caused by the interstitial atoms it is likely that the substitutional atoms do not have such significant influence of the critical stress for the plastic deformation as for the conventionally fabricated materials, which typically exhibited low YS. This is in agreement with the fact that the substitution of Nb by both Mo and Ta did not have an effect of the YS, although was previously reported for the cast alloys containing significantly lower concentrations of interstitial elements. In this case, the positive effect of substitution of Nb by Ta or Mo may result only from their effect on the martensitic transformation strain.

The addition of Mo and Ta to the base Ti-14Nb alloy resulted in the deterioration of the superelastic properties. This was associated with the increase of the β-phase stability and from the contribution of the twinning during the deformation. Twinning being a non-reversible mechanism caused the permanent deformation during the loading. The drop of the recoverable strain was moderate for Ti-14Nb-2Ta (1.7 ± 0.1%), since for this alloy both twinning and SIMT mechanism were observed. However, the value of 1.4 ± 0.1% for Ti-14Nb-2Mo was associated with the elasticity of the material.

## 4. Conclusions

The effect of the substitution of Nb by Mo and Ta and their addition on the mechanical and superelastic properties was investigated for the binary Ti-14Nb alloy prepared by the mechanical alloying and spark plasma sintering. Based on the obtained results the following conclusions may be drawn:

- The ternary alloying elements did not have a pronounced effect on the microstructure of the base Ti-14Nb alloy. It consists of the equiaxed β-phase grains, with the average size of about 30 µm, and precipitations of TiC formed during the sintering and annealing as a result of introduction of the WC-phase to the powders during the mechanical alloying.
- The impact of the substitution of Nb by Mo and Ta on the mechanical properties of the base Ti-14Nb alloy was slight, due a strong solid solution strengthening effect induced by the elevated amount of interstitial elements (oxygen and carbon). The fabricated materials exhibited high yield strength of about 800 MPa resulted from the occurrence of stress-induced martensitic transformation deformation mechanism.
- The addition of 2 at.% of Mo and Ta to base Ti-14Nb alloy resulted in a decrease of yield strength and increase of the plasticity of materials. This was associated with the increase of the β-phase stability and occurrence of {332} <113> twinning mechanism during the deformation of those materials. The effect of Mo addition on the β-phase stability was more pronounced because only the twinning was observed during the deformation of Ti-14Nb-2Mo, whereas in the case of Ti-14Nb-2Ta the twinning was observed together with the stress-induced martensitic transformation mechanism.
- The substitution of Nb by slight amounts of Mo or Ta did not exhibit a potential in enhancing the superelastic properties of the mechanically alloyed and sintered binary Ti-Nb alloys, due to a non-conventional superelastic behavior observed from them. Due to a negligible effect of those elements on the critical stress for plastic deformation in comparison to the interstitial atoms, their effect may be associated only with the slight changes in the martensitic transformation strain.

## Figures and Tables

**Table 1:** Mechanical properties of the investigated materials.

Alloy (at.%)	Ti-14Nb	Ti-8Nb-2Mo	Ti-12Nb-2Ta	Ti-14Nb-2Mo	Ti-14Nb-2Ta
Yield Strength (MPa)	790 ± 58	806 ± 36	792 ± 11	682 ± 15	766 ± 32
Compressive Strength (MPa)	1429 ± 81	1505 ± 85	1332 ± 49	1312 ± 80	1440 ± 88
Max. Compressive Strain (%)	20 ± 2	22 ± 1	17 ± 1	29 ± 2	25 ± 4

**Table 2:** Recoverable strains of the investigated materials.

Alloy (at.%)	Ti-14Nb	Ti-8Nb-2Mo	Ti-12Nb-2Ta	Ti-14Nb-2Mo	Ti-14Nb-2Ta
Recoverable strain (%)	2.1 ± 0.1	2.2 ± 0.1	2.2 ± 0.1	1.4 ± 0.1	1.7 ± 0.1

## Data Availability

The data presented in this study are available on request from the corresponding author.
